# (*S*)-2-(1*H*-Imidazol-1-yl)-3-phenyl­propanol

**DOI:** 10.1107/S1600536808004868

**Published:** 2008-02-27

**Authors:** Zuxing Yang, Siping Wei, Wenhai Wang, Hua Chen, Jingbo Lan

**Affiliations:** a Key Laboratory of Green Chemistry and Technology of the Ministry of Education, College of Chemistry, Sichuan University, Chengdu 610064, People’s Republic of China

## Abstract

In the title compound, C_12_H_14_N_2_O, the middle C atom in the propanol chain is a chiral center and possesses an *S* absolute configuration, according to the synthesis. In the crystal structure, inter­molecular O—H⋯N hydrogen bonds link the mol­ecules into a chain along the *b* axis.

## Related literature

For related literature, see: Bao *et al.* (2003 ▶); Baudequin *et al.* (2003 ▶); Lan *et al.* (2004 ▶); Matsuoka *et al.* (2006 ▶); Nair *et al.* (2004 ▶); Sambrook *et al.* (2005 ▶); Wang *et al.* (2007 ▶); You *et al.* (2001 ▶).
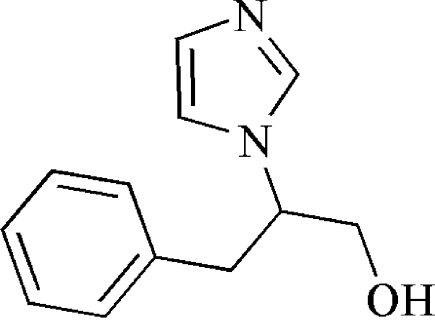


## Experimental

### 

#### Crystal data

C_12_H_14_N_2_O
*M* *_r_* = 202.25
Monoclinic, 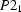

*a* = 8.021 (4) Å
*b* = 6.069 (3) Å
*c* = 11.629 (5) Å
β = 90.13 (5)°
*V* = 566.1 (5) Å^3^
*Z* = 2
Mo *K*α radiation
μ = 0.08 mm^−1^
*T* = 293 (2) K
0.40 × 0.33 × 0.23 mm

#### Data collection

Enraf–Nonius CAD-4 diffractometer
Absorption correction: none
1909 measured reflections
1146 independent reflections
826 reflections with *I* > 2σ(*I*)
*R*_int_ = 0.055
3 standard reflections every 200 reflections intensity decay: 1.2%

#### Refinement

*R*[*F* ^2^ > 2σ(*F* ^2^)] = 0.036
*wR*(*F* ^2^) = 0.090
*S* = 1.05
1146 reflections
142 parameters
1 restraint
H-atom parameters constrained
Δρ_max_ = 0.13 e Å^−3^
Δρ_min_ = −0.12 e Å^−3^

### 

Data collection: *DIFRAC* (Gabe & White, 1993 ▶); cell refinement: *DIFRAC*; data reduction: *NRCVAX* (Gabe *et al.*, 1989 ▶); program(s) used to solve structure: *SHELXS97* (Sheldrick, 2008 ▶); program(s) used to refine structure: *SHELXL97* (Sheldrick, 2008 ▶); molecular graphics: *ORTEPIII* (Burnett & Johnson, 1996 ▶); software used to prepare material for publication: *SHELXL97* and *PLATON* (Spek, 2003 ▶).

## Supplementary Material

Crystal structure: contains datablocks global, I. DOI: 10.1107/S1600536808004868/ez2119sup1.cif

Structure factors: contains datablocks I. DOI: 10.1107/S1600536808004868/ez2119Isup2.hkl

Additional supplementary materials:  crystallographic information; 3D view; checkCIF report

## Figures and Tables

**Table 1:** Hydrogen-bond geometry (Å, °)

*D*—H⋯*A*	*D*—H	H⋯*A*	*D*⋯*A*	*D*—H⋯*A*
O1—H1⋯N2^i^	0.82	1.98	2.802 (4)	177

Symmetry code: (i) 
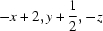
.

**Fig. 1.**
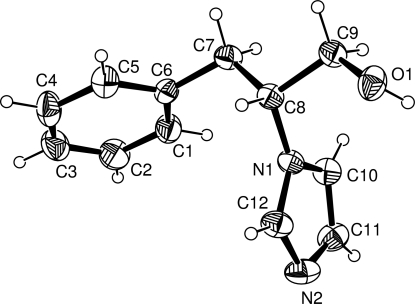
The molecular structure of (I), showing 30% probability displacement ellipsoids and the atomic numbering.

## supplementary crystallographic information

### Comment

Imidazoles are an important group in biological systems, and their derivatives have attracted widespread interest due to their application as precursors for imidazolium-based ionic liquids (Baudequin *et al.*, 2003), N-heterocyclic carbenes (Nair *et al.*, 2004) and molecular sensors (Sambrook *et al.*, 2005). We have focused our interest on the synthesis and applications of imidazole derivatives (Lan *et al.*, 2004; Wang *et al.*, 2007) and have reported several chiral cyclophanes and chiral molecular tweezers containing imidazole residues as receptors for the enantioselective recognition of amino acids or their derivatives (You *et al.*, 2001). Here, we report the crystal structure of the title compound, (I), which is a basic unit in the construction of chiral receptors and could be applied in the preparation of chair heterocyclic carbenes and ionic liquids.

In the structure of (I), the hydroxyl group and the imidazol-1-yl nitrogen atom are hydrogen bonded *via* an intermolecular O—H···N hydrogen bond as illustrated in Table 1 to result in the formation of a helical chain.

### Experimental

Since enantiopure amines are easily available and the chiral carbon has little risk of the racemization, they are usually applied for the preparation of chiral imidazole derivatives by cyclocondensation of ring fragments (Matsuoka *et al.*, 2006). The methyl 2-(1*H*-imidazol-1-yl)-3-phenylpropanoate was easily prepared according to a literature procedure (Bao *et al.*, 2003). Thereafter, NaBH_4_ (1.52 g, 40 mmol) was added to methyl 2-(1*H*-imidazol-1-yl)-3-phenylpropanoate (2.16 g, 10.0 mmol) in ethanol (150 ml) at 273 K over 30 min. The mixture was stirred at 333 K for another 20 h and then evaporated under vacuum. The residue was diluted with 20 ml saturated K_2_CO_3_ and extracted with 50 ml e thyl acetate. The organic layer was dried over anhydrous Na_2_SO_4_ and concentrated under reduced pressure. The resulting residue was purified by column chromatography on silica gel eluting with CH_2_Cl_2_/CH_3_OH (20/1, V/V), and then recrystallized from CH_2_Cl_2_ to give colorless crystals. Yield: 86%, mp 359–361 K, ^1^H NMR (300 MHz, CDCl_3_): δ 2.94–3.17 (dd, J=13.8 Hz, 7.7 Hz, 2H), 3.78–3.88 (m, 2H), 4.13–4.29 (m, 1H.), 6.88 (m, 2H), 7.02 (m, 2H), 7.28 (s, 1H), 7.03–7.26 (m, 5H). ^13^C NMR (75 MHz, CDCl_3_): δ 38.41, 62.26, 64.21, 117.61, 126.99, 128.59, 128.76, 128.90, 136.47, 137.10 p.p.m..

### Refinement

All H atoms were positioned geometrically and refined in the riding model approximation with C—H = 0.93, 0.97 Å and O—H = 0.82 Å. Since there is no atom heavier than oxygen it was not possible to determine the absolute structure exactly. However, the chiral carbon does not directly participate in the cyclocondensation in this reaction (Matsuoka *et al.*, 2006). Herein, the desired product (*S*)-2-(1*H*-imidazol-1-yl)-3-phenylpropanol was obtained starting from (*S*)-2-amino-3-phenylpropanoic acid, whose absolute configuration (S) is consistent with the starting material.

### Crystal data

**Table:**

C_12_H_14_N_2_O	*F*_000_ = 216
*M**_r_* = 202.25	*D*_x_ = 1.186 Mg m^−^^3^
Monoclinic, *P*2_1_	Mo *K*α radiation λ = 0.71073 Å
Hall symbol: P 2yb	Cell parameters from 26 reflections
*a* = 8.021 (4) Å	θ = 4.6–7.8º
*b* = 6.069 (3) Å	µ = 0.08 mm^−^^1^
*c* = 11.629 (5) Å	*T* = 293 (2) K
β = 90.13 (5)º	Block, colorless
*V* = 566.1 (5) Å^3^	0.40 × 0.33 × 0.23 mm
*Z* = 2	

### Data collection

**Table:**

Enraf–Nonius CAD-4 diffractometer	*R*_int_ = 0.055
Radiation source: fine-focus sealed tube	θ_max_ = 25.4º
Monochromator: graphite	θ_min_ = 1.8º
*T* = 293(2) K	*h* = −9→0
ω/2θ scans	*k* = −7→7
Absorption correction: none	*l* = −13→14
1909 measured reflections	3 standard reflections
1146 independent reflections	every 200 reflections
826 reflections with *I* > 2σ(*I*)	intensity decay: 1.2%

### Refinement

**Table:**

Refinement on *F*^2^	Secondary atom site location: difference Fourier map
Least-squares matrix: full	Hydrogen site location: inferred from neighbouring sites
*R*[*F*^2^ > 2σ(*F*^2^)] = 0.036	H-atom parameters constrained
*wR*(*F*^2^) = 0.090	*w* = 1/[σ^2^(*F*_o_^2^) + (0.0335*P*)^2^ + 0.0435*P*] where *P* = (*F*_o_^2^ + 2*F*_c_^2^)/3
*S* = 1.05	(Δ/σ)_max_ < 0.001
1146 reflections	Δρ_max_ = 0.13 e Å^−^^3^
142 parameters	Δρ_min_ = −0.12 e Å^−^^3^
1 restraint	Extinction correction: SHELXL97 (Sheldrick, 2008), Fc^*^=kFc[1+0.001xFc^2^λ^3^/sin(2θ)]^-1/4^
Primary atom site location: structure-invariant direct methods	Extinction coefficient: 0.045 (8)

### Special details

**Table:**

Experimental. In the crystal structure, there is not any heavy atom than silicon, so we can't get the absolute structure exactly. However, the chiral carbon does not directly participate in the cyclocondensation in this reaction (Matsuoka *et al.*, Tetrahedron. 2006, 62, 8199–8206). From starting material (*S*)-2-amino-3-phenylpropanoic acid, it give (*S*)-2-(1*H*-imidazol-1-yl)-3-phenylpropanol as product, whose absolute configuration (S) is consistent with the absolute structure characterized by X-ray structure analysis.
Geometry. All e.s.d.'s (except the e.s.d. in the dihedral angle between two l.s. planes) are estimated using the full covariance matrix. The cell e.s.d.'s are taken into account individually in the estimation of e.s.d.'s in distances, angles and torsion angles; correlations between e.s.d.'s in cell parameters are only used when they are defined by crystal symmetry. An approximate (isotropic) treatment of cell e.s.d.'s is used for estimating e.s.d.'s involving l.s. planes.
Refinement. Refinement of *F*^2^ against ALL reflections. The weighted *R*-factor *wR* and goodness of fit *S* are based on *F*^2^, conventional *R*-factors *R* are based on *F*, with *F* set to zero for negative *F*^2^. The threshold expression of *F*^2^ > σ(*F*^2^) is used only for calculating *R*-factors(gt) *etc*. and is not relevant to the choice of reflections for refinement. *R*-factors based on *F*^2^ are statistically about twice as large as those based on *F*, and *R*- factors based on ALL data will be even larger.

### Fractional atomic coordinates and isotropic or equivalent isotropic displacement parameters (Å^2^)

**Table:**

	*x*	*y*	*z*	*U*_iso_*/*U*_eq_	
O1	0.6488 (3)	0.9519 (5)	−0.06637 (15)	0.0601 (7)	
H1	0.7178	1.0460	−0.0835	0.086 (15)*	
N1	0.8556 (3)	0.8538 (4)	0.13967 (17)	0.0418 (6)	
N2	1.1263 (3)	0.7863 (6)	0.1257 (2)	0.0651 (9)	
C1	0.7787 (4)	0.7337 (7)	0.4142 (2)	0.0628 (10)	
H1B	0.8315	0.8693	0.4069	0.073 (4)*	
C2	0.8278 (4)	0.5915 (8)	0.5001 (3)	0.0747 (12)	
H2	0.9137	0.6313	0.5497	0.073 (4)*	
C3	0.7501 (5)	0.3908 (7)	0.5128 (3)	0.0725 (12)	
H3	0.7824	0.2955	0.5714	0.073 (4)*	
C4	0.6247 (5)	0.3324 (7)	0.4383 (2)	0.0680 (10)	
H4	0.5716	0.1970	0.4464	0.073 (4)*	
C5	0.5773 (4)	0.4746 (6)	0.3512 (2)	0.0565 (9)	
H5	0.4935	0.4324	0.3003	0.073 (4)*	
C6	0.6522 (3)	0.6781 (6)	0.3385 (2)	0.0456 (8)	
C7	0.5912 (3)	0.8393 (6)	0.2488 (2)	0.0498 (8)	
H7A	0.6073	0.9875	0.2780	0.046 (4)*	
H7B	0.4723	0.8176	0.2383	0.046 (4)*	
C8	0.6752 (3)	0.8225 (6)	0.1318 (2)	0.0431 (7)	
H8	0.6550	0.6738	0.1021	0.040 (7)*	
C9	0.5963 (3)	0.9848 (6)	0.0484 (2)	0.0517 (8)	
H9A	0.4760	0.9703	0.0520	0.046 (4)*	
H9B	0.6248	1.1335	0.0719	0.046 (4)*	
C10	0.9385 (4)	1.0364 (6)	0.1796 (2)	0.0514 (9)	
H1A	0.8907	1.1653	0.2075	0.073 (4)*	
C11	1.1038 (4)	0.9919 (7)	0.1705 (2)	0.0607 (10)	
H11	1.1892	1.0874	0.1917	0.073 (4)*	
C12	0.9738 (4)	0.7106 (6)	0.1088 (2)	0.0542 (8)	
H12	0.9510	0.5720	0.0785	0.073 (4)*	

### Atomic displacement parameters (Å^2^)

**Table:**

	*U*^11^	*U*^22^	*U*^33^	*U*^12^	*U*^13^	*U*^23^
O1	0.0642 (15)	0.0700 (16)	0.0462 (11)	−0.0137 (15)	−0.0054 (9)	0.0087 (11)
N1	0.0410 (12)	0.0430 (15)	0.0414 (11)	0.0035 (14)	−0.0051 (9)	−0.0040 (11)
N2	0.0411 (16)	0.088 (3)	0.0659 (16)	0.0082 (17)	−0.0038 (12)	−0.0079 (17)
C1	0.057 (2)	0.066 (3)	0.0656 (19)	−0.009 (2)	−0.0107 (15)	0.0151 (19)
C2	0.065 (2)	0.093 (3)	0.066 (2)	−0.001 (2)	−0.0198 (18)	0.017 (2)
C3	0.085 (3)	0.075 (3)	0.058 (2)	0.016 (3)	0.0044 (18)	0.0208 (19)
C4	0.101 (3)	0.050 (2)	0.0522 (17)	0.001 (2)	0.0205 (18)	0.0072 (19)
C5	0.074 (2)	0.050 (2)	0.0459 (15)	−0.004 (2)	0.0051 (14)	−0.0072 (16)
C6	0.0442 (16)	0.050 (2)	0.0429 (15)	−0.0001 (17)	0.0058 (12)	0.0041 (14)
C7	0.0440 (16)	0.056 (2)	0.0496 (15)	0.0050 (18)	−0.0027 (12)	−0.0008 (16)
C8	0.0419 (15)	0.0454 (19)	0.0419 (13)	0.0001 (16)	−0.0061 (11)	−0.0023 (14)
C9	0.0467 (17)	0.056 (2)	0.0527 (15)	0.0023 (19)	−0.0078 (12)	0.0072 (16)
C10	0.0512 (19)	0.051 (2)	0.0519 (16)	−0.0015 (18)	−0.0039 (14)	−0.0075 (15)
C11	0.0465 (19)	0.081 (3)	0.0544 (16)	−0.007 (2)	−0.0072 (13)	−0.002 (2)
C12	0.056 (2)	0.060 (2)	0.0471 (15)	0.012 (2)	−0.0004 (13)	−0.0109 (16)

### Geometric parameters (Å, °)

**Table:**

O1—C9	1.415 (3)	C4—H4	0.9300
O1—H1	0.8200	C5—C6	1.382 (5)
N1—C12	1.336 (4)	C5—H5	0.9300
N1—C10	1.372 (4)	C6—C7	1.511 (4)
N1—C8	1.462 (3)	C7—C8	1.522 (3)
N2—C12	1.321 (4)	C7—H7A	0.9700
N2—C11	1.364 (5)	C7—H7B	0.9700
C1—C2	1.377 (5)	C8—C9	1.519 (4)
C1—C6	1.383 (4)	C8—H8	0.9800
C1—H1B	0.9300	C9—H9A	0.9700
C2—C3	1.376 (6)	C9—H9B	0.9700
C2—H2	0.9300	C10—C11	1.358 (4)
C3—C4	1.373 (5)	C10—H1A	0.9300
C3—H3	0.9300	C11—H11	0.9300
C4—C5	1.384 (5)	C12—H12	0.9300
			
C9—O1—H1	109.5	C8—C7—H7A	108.4
C12—N1—C10	105.8 (2)	C6—C7—H7B	108.4
C12—N1—C8	127.0 (3)	C8—C7—H7B	108.4
C10—N1—C8	127.2 (3)	H7A—C7—H7B	107.5
C12—N2—C11	104.6 (3)	N1—C8—C9	111.5 (2)
C2—C1—C6	121.1 (4)	N1—C8—C7	112.0 (2)
C2—C1—H1B	119.4	C9—C8—C7	110.0 (2)
C6—C1—H1B	119.4	N1—C8—H8	107.7
C3—C2—C1	120.3 (3)	C9—C8—H8	107.7
C3—C2—H2	119.9	C7—C8—H8	107.7
C1—C2—H2	119.9	O1—C9—C8	112.7 (3)
C4—C3—C2	119.5 (3)	O1—C9—H9A	109.0
C4—C3—H3	120.3	C8—C9—H9A	109.0
C2—C3—H3	120.3	O1—C9—H9B	109.0
C3—C4—C5	120.1 (4)	C8—C9—H9B	109.0
C3—C4—H4	120.0	H9A—C9—H9B	107.8
C5—C4—H4	120.0	C11—C10—N1	106.6 (3)
C6—C5—C4	121.1 (3)	C11—C10—H1A	126.7
C6—C5—H5	119.4	N1—C10—H1A	126.7
C4—C5—H5	119.4	C10—C11—N2	110.0 (3)
C5—C6—C1	117.9 (3)	C10—C11—H11	125.0
C5—C6—C7	120.8 (3)	N2—C11—H11	125.0
C1—C6—C7	121.2 (3)	N2—C12—N1	113.0 (3)
C6—C7—C8	115.5 (3)	N2—C12—H12	123.5
C6—C7—H7A	108.4	N1—C12—H12	123.5

### Hydrogen-bond geometry (Å, °)

**Table:**

*D*—H···*A*	*D*—H	H···*A*	*D*···*A*	*D*—H···*A*
O1—H1···N2^i^	0.82	1.98	2.802 (4)	177

Symmetry codes: (i) −*x*+2, *y*+1/2, −*z*.
